# Crosstalk in competing endogenous RNA networks reveals new circular RNAs involved in the pathogenesis of early HIV infection

**DOI:** 10.1186/s12967-018-1706-1

**Published:** 2018-11-29

**Authors:** Yue Zhang, Hui Zhang, Minghui An, Bin Zhao, Haibo Ding, Zining Zhang, Youwen He, Hong Shang, Xiaoxu Han

**Affiliations:** 1 NHC Key Laboratory of AIDS Immunology (China Medical University), Department of Laboratory Medicine, The First Affiliated Hospital of China Medical University, No 155, Nanjing North Street, Heping District, Shenyang, 110001 Liaoning China; 2 Key Laboratory of AIDS Immunology of Liaoning Province, The First Affiliated Hospital of China Medical University, Shenyang, 110001 China; 3 Key Laboratory of AIDS Immunology, Chinese Academy of Medical Sciences, Shenyang, 110001 China; 4 Collaborative Innovation Center for Diagnosis and Treatment of Infectious Diseases, 79 Qingchun Street, Hangzhou, 310003 China; 5 Department of Immunology, Medical Center of Duke University, Durham, NC USA

**Keywords:** HIV-1, circRNA, ceRNA, Viral replication

## Abstract

**Background:**

The events in early HIV infection (EHI) are important determinants of disease severity and progression rate to AIDS, but the mechanisms of pathogenesis in EHI have not been fully understood. Circular RNAs (circRNAs) have been verified as “microRNA sponges” that regulate gene expression through competing endogenous RNA (ceRNA) networks, but circRNA expression profiles and their contribution to EHI pathogenesis are still unclear.

**Methods:**

Two different libraries were constructed with RNA from human peripheral blood mononuclear cells from 3 HARRT-naive EHI patients and 3 healthy controls (HCs). The complete transcriptomes were sequenced with RNA sequencing (RNA-Seq) and miRNA sequencing (miRNA-Seq). The differentially expressed (DE) RNAs were validated with RT-qPCR. The circRNA profile and circRNA-associated-ceRNA network in EHI were analyzed with the integrated data of RNA-Seq and miRNA-Seq. Gene ontology (GO) analysis was used to annotate the circRNAs involved in the circRNA-associated-ceRNA networks.

**Results:**

A total of 1365 circRNAs, 30 miRNAs, and 2049 mRNAs were differentially expressed between HARRT-naive EHI patients and HCs. A ceRNA network was constructed with 516 DE circRNAs and 903 DE mRNAs that shared miR response elements with 21 DE miRNAs. GO analysis demonstrated the multiple roles of the circRNAs enriched in EHI with circRNA-associated-ceRNA networks, such as immune response, inflammatory response and defense responses to virus, 67 circRNAs were revealed to be potentially involved in HIV-1 replication through regulating the expression of CCNK, CDKN1A and IL-15.

**Conclusions:**

This study, for the first time, revealed a large circRNA profile and complex pathogenesis roles of circRNAs in EHI. A group of enriched circRNAs and associated circRNA-associated-ceRNA networks might contribute to HIV replication regulation and provide novel potential targets for both the pathogenesis of EHI and antiviral therapy.

**Electronic supplementary material:**

The online version of this article (10.1186/s12967-018-1706-1) contains supplementary material, which is available to authorized users.

## Background

Early HIV infection (EHI) represents a stage that extends for approximately 6 months post infection, which is believed to be the key stage determining the subsequent disease progression rate to AIDS. EHI is characterized by viral replication dramatically increasing to a peak level and then rapidly declining to a stable level (viral set point), intense antiviral immune response and immune injury [1–6]. Studies of EHI will deepen the understanding of the early responses of hosts to try to control HIV-1 replication and contribute to long-term clinical outcome predictions.

Genome-wide transcriptome studies of EHI in HIV-infected patients and non-human primate models identified that both protein coding genes and non-coding genes were involved in the pathogenesis of EHI, including genes that regulate innate immunity, cell proliferation, cell cycle and the immune response [7–10]. In HIV-infected patients, a set of 12 genes were reported to be associated with viral load and predicted the viral set point, with the hub of hydrogen peroxide, IFNG, TGFB1 and TNF [9]. Multiple host proteins, such as APOBEC3G, TRIM5α, MX2, BST2 and SAMHD1, were extensively studied and demonstrated to be key regulators of viral replication in different stages of the HIV life cycle in certain host cells [11–18]. Long non-coding RNAs (lncRNAs), such as NEAT1 and NRON, are precisely regulated during the HIV-1 life cycle and were also reported to be host factors utilized by HIV for infection and persistence [19–21], Moreover, many microRNAs (miRNAs) are believed to play important roles in post-transcriptional regulation and contribute to both the pathogenesis and clinical outcome of HIV infection, according to several miRNA profiling studies [22–24]. For example, miR-198 was reported to restrict HIV-1 replication in monocytes through repression of cyclin T1 [25]. In another example, an miRNA profiling study by our group identified 5 miRNAs (miR-31, miR-99a, miR-200c, miR-503 and miR-526a) from EHI that are associated with a rapid progression of HIV infection with a predictive value of 94%. Moreover, overexpression of miR-31 in primary human T cells promoted their survival [10].

Different from lncRNAs and miRNAs, circular RNA (circRNA) is a novel class of non-coding RNA without free 3′ or 5′ ends like traditional linear RNA, but instead, they form covalently closed-loop structures [26, 27]. The majority of circRNA molecules contain dozens of conserved sequences matching miRNAs and thus can bind to multiple miRNAs and inhibit their function through acting as “miRNA sponges” [28], according to the competing endogenous RNA (ceRNA) hypothesis of circRNA [29], which has been supported by accumulating evidence in many diseases [30, 31].

In bladder carcinoma, circRNA-MYLK directly binds to miR-29a and acts as a ceRNA, which contributes to epithelial–mesenchymal transition and bladder carcinoma development through activating VEGFA/VEGFR2 and its downstream Ras/ERK signaling pathway [32]. Moreover, two recent studies have connected circRNAs to innate immune pathways in viral infection [33, 34]. One study reported that exogenous circRNAs co-aggregate with the nucleic acid sensor RIG-I, induce innate immunity genes and promote protection against viral infection [34]. Another study demonstrated viral infection can partially decrease circRNA expression by binding to NF90/NF110, a double-stranded RNA-binding domain containing immune factors that are then released from circRNP complexes and induce the nuclear export of NF90/NF110 to the cytoplasm [33]. In EHI, both high level viral replication and strong immune responses occur in vivo; however, the role of circRNAs in the pathogenesis of EHI requires further investigation.

In this study, we aimed to explore the expression profiles of circRNAs and the potential roles of circRNAs in the pathogenesis of EHI through two integrated omics data of RNA sequencing (RNA-Seq) and miRNA sequencing (miRNA-Seq) on peripheral blood mononuclear cells (PBMCs) from HARRT-naïve EHI patients and 3 healthy controls (HCs). CircRNA-associated-ceRNA networks were constructed to reveal the differentially expressed (DE) circRNAs involved in pathways. This study, for the first time, revealed a large circRNA profile and complex pathogenesis roles of circRNAs in EHI. A group of enriched circRNAs and associated circRNA-associated-ceRNA networks might contribute to HIV replication regulation and provides novel potential targets for both the pathogenesis of EHI and antiviral therapy.

## Methods

### Patients

A total of 33 HARRT-naive EHI patients, 10 HARRT-naive chronic HIV infection (CHI) patients and 17 HCs were enrolled in this study. RNA-Seq and miRNA-Seq were performed on samples from 3 EHI patients and 3 HCs. RT-qPCR validation was performed on 19 EHI,10 CHI and 9 HCs for DE circRNAs, 11 EHI and 5 HCs for DE mRNAs, and 17 EHI and 8 HCs for DE miRNAs. The EHI patients in this study were recruited from a large-scale prospective cohort of HIV-negative Men who have sex with men, who were followed up every 1.5–3 months to test HIV status with Elecsys HIV combi PT assay (Roche), HIV 1 + 2 Antibody Detection Kit (MP Biomedical Asia Pacific Private Ltd). and pooled nucleic acid amplification testing (Roche). The time of HIV infection was estimated according to the published standards of acute HIV infection [35]. In brief, the infection time was estimated as 14 days for case with HIV Ag/Ab(−) and pooling NAAT(+), and 30 days with HIV Ag/Ab(+), West Blot (undetermined) and pooling NAAT(+), or the middle point between the last HIV Ag/Ab(−) NAAT(−) and first HIV Ag/Ab(+). The EHI patients were defined as patients were within 180 days since diagnosed as HIV infection, and the CHI patients were defined as patients were beyond 180 days since diagnosed as HIV infection. Both EHI and CHI patients did not receive antiretroviral therapy at the time this study was conducted. For patient EHI-1, EHI-2, and EHI-3 that enrolled in RNA-Seq and miRNA-Seq were firstly diagnosed as HIV infection at 49 days, 29 days and 30 days post infection, respectively, and the samples collected at 159 days, 139 days and 139 days post infection for patients EHI-1, EHI-2 and EHI3 respectively. Characteristics of the subjects enrolled in the study are summarized in Additional file 1: Table S1. PBMCs were obtained from EHI patients, CHI patients and HCs by Ficoll–Hypaque (GE Healthcare) density gradient centrifugation, then cryopreserved in fetal calf serum (Gibco) supplemented with 10% DMSO (Sigma) and stored in liquid nitrogen until RNA extraction. The study was reviewed and approved by the local ethics review committee. All participants provided written informed consent prior to research participation.

### RNA extraction and quality control

Total RNA was extracted from 5 M PBMCs with TRIzol reagent (Life Technologies), residual DNA was removed with TurboDnase (Life Technologies), and then the quality of the RNA was evaluated with Qubit (Life Technologies) measurement followed by a Bioanalyzer (Agilent Technologies) evaluation.

### RNA library construction and sequencing of RNA

RiboMinus™ (Life Technologies) was used to enrich the whole spectrum of RNA transcripts by selectively depleting ribosomal RNA molecules (rRNA). After cDNA synthesis, DNA from each sample was clustered and sequenced on an Illumina HiSeq 2500 sequencing system (Illumina) in 100-bp paired-end reads following the manufacturer’s instructions.

### miRNA library construction and miRNA sequencing

NEBNext Multiplex Small RNA Library Prep Set for Illumina (New England Biolabs) was used to prepare the miRNA sequencing library. The miRNA sequencing libraries were denatured as single-stranded DNA molecules, captured on Illumina flow cells, amplified in situ as clusters and finally sequenced for 50 cycles on a HiSeq 4000 sequencing system (Illumina) following the manufacturer’s instructions.

### RNA-Seq analysis

First, the raw reads were aligned to the human reference genome (UCSC hg19) with hisat2 (v2.0.4) software (http://ccb.jhu.edu/software/hisat2/index.shtml). Then, using the cuffdiff software (part of cufflinks), the FPKM (fragments per kilobase of exon per million fragments mapped) [36] were obtained under the guidance of the gtf gene annotation file as the expression profiles of mRNA, and fold change and P-values between the two group samples were calculated based on FPKM. Second, the raw reads were aligned to the human reference genome with STAR software [37], and circRNA detection and identification was performed using DCC software [38]. To label the circRNAs, we used the CircBase database [39] and circ2Trait disease database [40], and edgeR software [41] was used to normalize the data and perform DE circRNA analysis.

### miRNA Analysis

We used cutadapt software (v1.9.3) to trim the adaptor sequences, and the adaptor-trimmed-reads (≥ 15 nt) were left. Then, trimmed reads of all samples were pooled, and novel miRNA predictions were performed using miRDeep2 software (v2.0.0.5) [42]. The trimmed reads of each sample were aligned to the pooled human pre-miRNA databases (known pre-miRNA from miRBase (http://www.mirbase.org) plus the newly predicted pre-miRNAs) using Novoalign software (v3.02.12) (http://www.novocraft.com/main/index.php). The numbers of tags on each mature miRNA were defined as the original expression levels of the miRNA, and the TPM [43] (tag counts per million aligned miRNAs) approach was used to normalize the read counts. P-values and fold changes between the two groups of samples were calculated, and DE miRNAs were screened.

### Real-time quantitative PCR validation

To validate the accuracy of the sequencing results, real-time quantitative PCR (RT-qPCR) with SYBR green analysis was used. For DE mRNAs and circRNAs validation, total RNA was extracted using TRIzol reagent (Life Technologies). For DE miRNAs validation, total RNA was isolated miRNeasy Micro kit (Qiagen). The circRNAs and mRNAs were reverse transcribed using a Primpscript^®^RT reagent kit (TAKARA), and miRNAs were reverse transcribed using a Mir-X™ miRNA First Strand Synthesis Kit (TAKARA) following the manufacturer’s instructions. RT-qPCR was performed with SYBR^®^ Premix Ex Taq™ II (TAKARA). RPLP0, GAPDH and U6 were used as endogenous control for mRNA, circRNA and miRNA RT-qPCR, respectively. The expression levels of mRNA, circRNA and miRNA expression were calculated based on the change in cycling threshold with the method of 2^−ΔΔCt^ [44].

### ceRNA network analysis

TargetScan (http://www.targetscan.org) was used to predict the miRNA binding seed sequence sites. The DE circRNAs and mRNAs that shared the same miRNA binding site represented circRNA-miRNA-mRNA interactions. The ceRNA network was constructed with RNAs meeting the following standards: (1) differentially expressed; (2) the expression of miRNA and circRNA and miRNA and mRNA were inversely regulated; (3) ‘7mer’ (nucleotides 2–8 without mismatches or Wobble pairing) and ‘8mer’ [an exact match to positions 2–8 of the mature miRNA (the seed + position 8) followed by an ‘A’] seed pairing for each individual DE miRNA against mRNAs and circRNAs [45] to improve the reliability of prediction; and (4) miRNAs had records in miRBase. Cytoscape software (v3.5.1) [46] was used to display the ceRNA network.

### Gene ontology analysis

Gene ontology analysis of the DE mRNAs and the circRNA-miRNA-enriched genes identified in this study was performed with the Database for Annotation, Visualization and Integrated Discovery (DAVID, Version 6.8 Beta) (http://david.abcc.ncifcrf.gov/) [47]. P value less than 0.05 was considered to indicate a statistically significant difference.

### Statistical analysis

GraphPad Prism 5 (GraphPad Software) was used to perform RT-qPCR statistical analysis. The non-parametric Mann–Whitney U test was used to compare between-group distributions. A two-tailed P value less than 0.05 was considered to be statistically significant.

## Results

### Identification of DE mRNAs between HARRT-naïve EHI patients and HCs

Firstly, we analyzed the profiling of mRNA of PBMCs from 3 HARRT-naïve EHI patients and 3 HCs. A total of 20,315 mRNA transcripts were identified, among which 2049 mRNAs were differentially expressed between the two groups with a fold change above 1.5 (*P* ≤ 0.05), including 673 up-regulated mRNAs and 1376 down-regulated mRNAs. Gene ontology (GO) analysis was carried out to investigate the function of the DE mRNAs. The top five significantly enriched GO terms were SRP-dependent cotranslational protein targeting to membrane (GO:0006614), nuclear-transcribed mRNA catabolic process, nonsense-mediated decay (GO:0000184), viral transcription (GO:0019083), nucleosome assembly (GO:0006334) and immune response (GO:0006955) (Additional file 2: Table S2).

### Identification of DE circRNAs between HARRT-naïve EHI patients and HCs

**Fig. 1:**
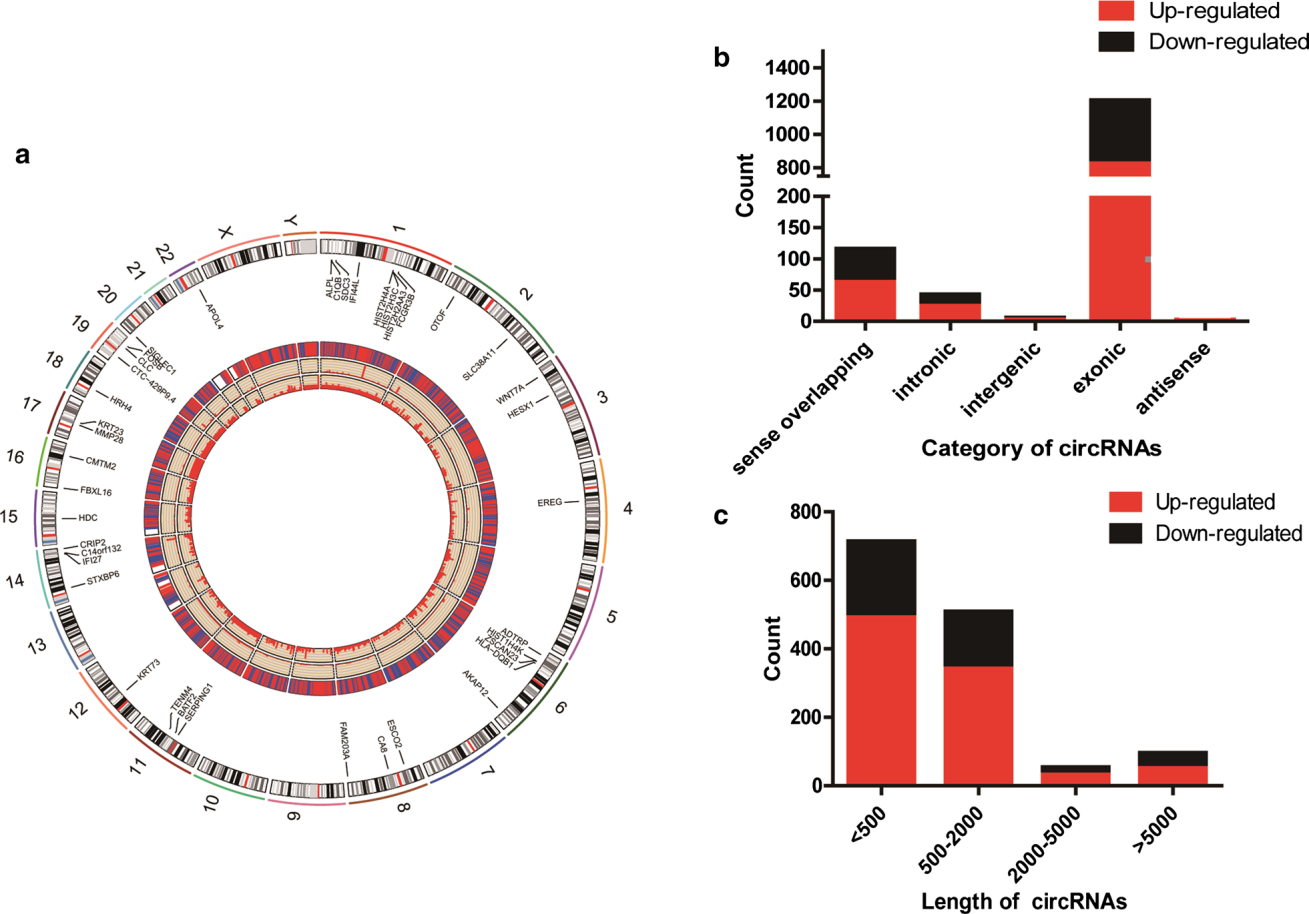
The Profiling and Characteristics of mRNAs and circRNAs in EHI. **a** Circos plot showing the mRNAs and circRNAs on human chromosomes. From the outside in, the first layer of the Circos plot is a chromosome map of the human genome; black and white bars are chromosome cytobands, and red bars represent centromeres. The second layer shows the 20 most significantly up-regulated and 20 most significantly down-regulated mRNAs. All DE mRNAs and circRNAs are marked in red and blue in the third layer. The fourth and fifth layers show the fold change of all DE mRNAs and circRNAs. **b** The counts of DE circRNAs based on their categories. **c** The distribution of the DE circRNAs based on the length of nucleic acids

From the RNA-Seq data, a total of 15,145 circRNA transcripts were identified, among which 1365 circRNAs were differentially expressed between the two groups with a fold change above 2.0 (P ≤ 0.05), including 912 up-regulated circRNAs and 453 down-regulated circRNAs. The coding sequences for the DE circRNAs were located in 22 autosomes and the X and Y chromosome separately without aggregation (Fig. 1a). Five categories of circRNAs were detected, including sense overlapping circRNAs, intronic circRNAs, intergenic circRNAs, exonic circRNAs and antisense circRNAs, with constituent ratios of 8.42% (115/1365), 3.08% (42/1365), 0.37% (5/1365), 88.06% (1202/1365) and 0.07% (1/1365), respectively (Fig. 1b). The length of 89.30% (1219/1365) of the circRNAs was less than 2000 nt and the median length was above 500 nt (Fig. 1c). The number of up-regulated circRNAs was greater than that of the down-regulated circRNAs in every circRNA category and length group.

### Identification of DE miRNAs between HARRT-naïve EHI patients and HCs

We further determined the miRNA expression profile using microRNA sequencing (miRNA-Seq) with the RNAs from the same PBMC samples that were used in the mRNA and circRNA transcriptome study. A total of 1304 mature miRNAs were detected, including 839 with records in miRBase and 465 (35.66% of 1304) without records in miRBase, defined as potential novel miRNAs. However, only 30 miRNAs were differentially expressed between the two groups with fold changes above 1.5 (*P* ≤ 0.05), including 12 up-regulated miRNAs (7 known and 5 novel) and 18 down-regulated miRNAs (14 known and 4 novel). Among these DE miRNAs, miR-novel-chr21_21352, miR-101-3p, and miR-31-5p were the top three miRNAs that were the most significantly differentially expressed in EHI (Additional file 3: Table S3).

### Real-time quantitative PCR validation

**Fig. 2:**
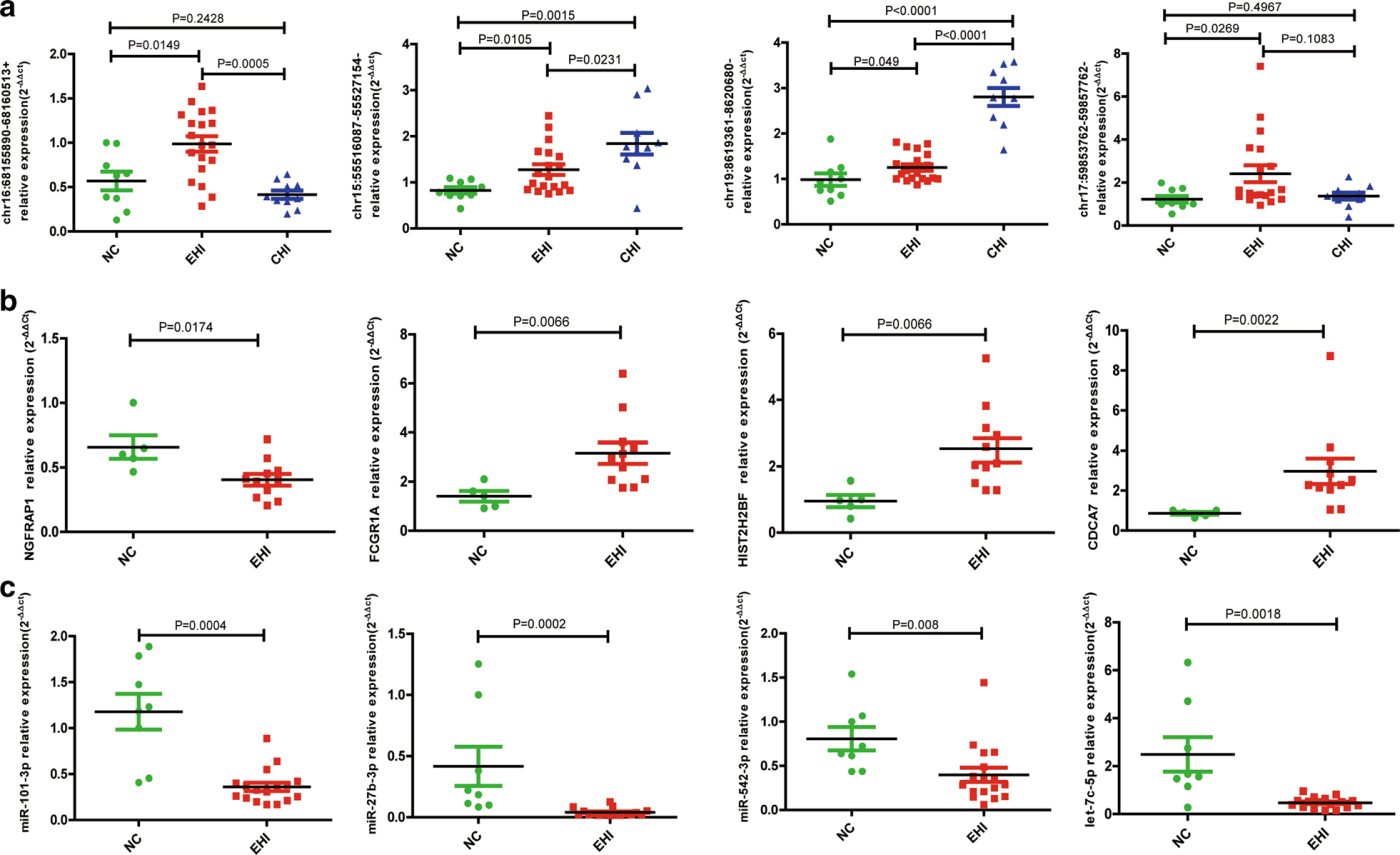
RNA-Seq and miRNA-Seq validated by RT-qPCR. **a** Validation of four DE circRNAs. **b** Validation of four DE mRNAs. **c** Validation of four DE miRNAs

To validate the DE RNA profiling, twelve DE transcripts were randomly selected and validated with RT-qPCR, including 4 circRNAs, 4 miRNAs, and 4 mRNAs. As shown in Fig. 2, the results of RT-qPCR were highly consistent with the results of RNA-Seq and miRNA-Seq for all of the twelve DE transcripts (Fig. 2a–c). To further explore whether the changes of targeting circRNAs expression are specific to EHI, we also tested the expression level of 4 circRNAs among 10 CHI patients. It was interesting to note that chr16:68155890−68160513+ and chr17:59853762−59857762− were up-regulated in EHI group but not CHI group compared with HC group, which suggested these two circRNAs might be involved in processes specific to EHI stage. While the expression level of chr15:55516087−55527154− and chr19:8619361−8620680− in the CHI patients were higher than those in EHI group, which suggested that these circRNAs might be associated with HIV infection status or disease progression (Fig. 2a).

### circRNA–miRNA–mRNA ceRNA networks

**Fig. 3:**
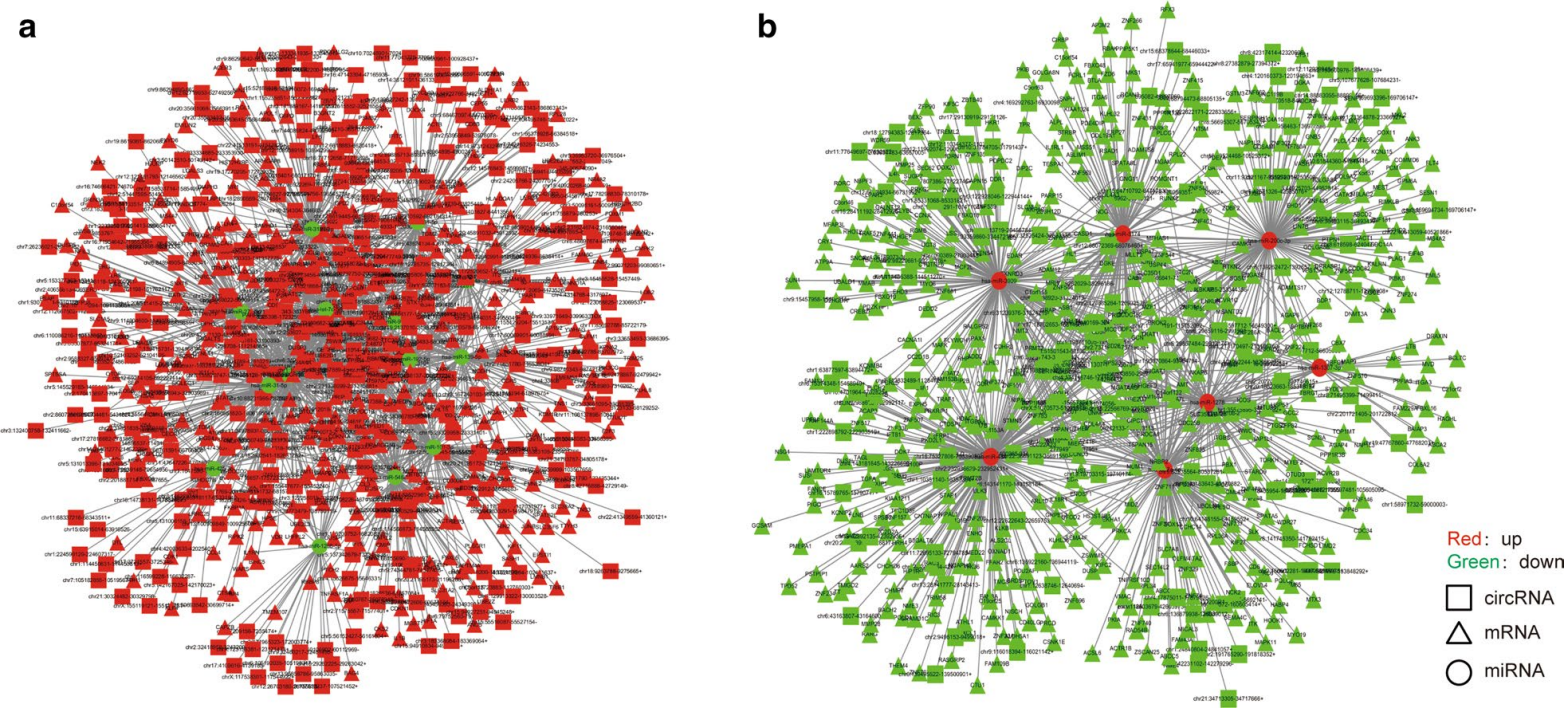
circRNA-associated-ceRNA networks in EHI. The ceRNA networks were based on circRNA-miRNA and miRNA-mRNA interactions. Shape corresponds to molecule type (circRNAs as squares, mRNAs as triangles, miRNAs as circles), colour corresponds to dysregulation (red as up-regulated, green as down-regulated). **a** circRNA (up in EHI)-miRNA (down in EHI)-mRNA (up in EHI). **b** circRNA (down in EHI)-miRNA (up in EHI)-mRNA (down in EHI)

To explore the potential biological process of DE circRNAs involvement, we constructed a circRNA–miRNA–mRNA network with DE mRNAs, circRNAs and miRNAs, based on the hypothesis that circRNAs might regulate the expression of mRNAs through sharing a common binding site of miR response elements (MREs). The standards used for circRNA, miRNA and mRNA selection in the ceRNA network analyses are listed in the Materials. A ceRNA network was constructed with 516 DE circRNAs and 903 DE mRNAs that shared MREs of 21 DE miRNAs. The ceRNA network included two cases (Fig. 3a, b): one included 366 up-regulated circRNAs, 14 down-regulated miRNAs and 378 up-regulated mRNAs, while the other included 150 down-regulated circRNAs, 7 up-regulated miRNAs and 525 down-regulated mRNAs.

**Fig. 4:**
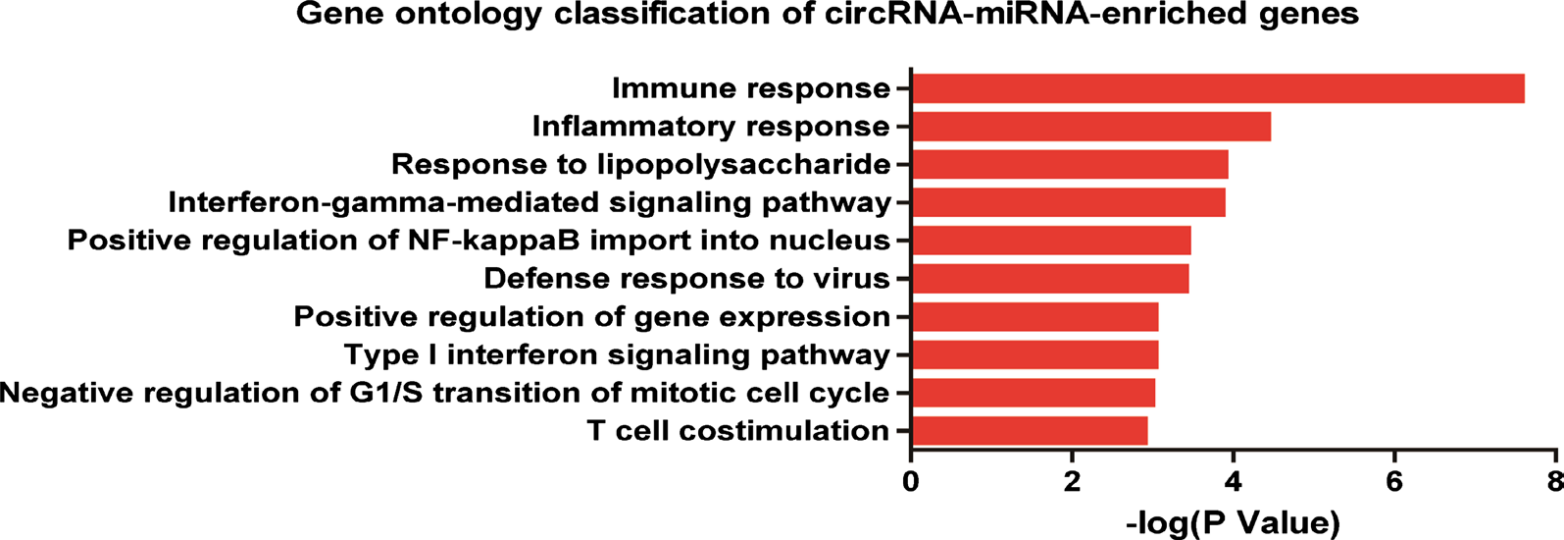
Gene ontology classifcations of the predicted targeting mRNAs of the circRNA-associated-ceRNA networks. The 10 most significantly enriched GO terms of the predicted targeting mRNAs of the circRNA-associated-ceRNA networks. P < 0.05 was used as the threshold for GO analysis

In order to further explore the function of enriched circRNAs and corresponding ceRNA networks in EHI, GO analysis was performed on the predicted targeting mRNAs among each of the circRNA-associated-ceRNA networks. It was shown that 107 terms were significantly enriched among targeting mRNAs. The top 10 enriched terms were immune response (GO:0006955), inflammatory response (GO:0006954), response to lipopolysaccharide (GO:0032496), interferon-gamma-mediated signaling pathway (GO:0060333), positive regulation of NF-kappaB import into nucleus (GO:0042346), defense response to virus (GO:0051607), positive regulation of gene expression (GO:0010628), type I interferon signaling pathway (GO:0060337), negative regulation of G1/S transition of mitotic cell cycle (GO:2000134) and T cell costimulation (GO:0031295) (Fig. 4).

### HIV-1 replication associated ceRNA network

From the top 10 circRNA–miRNA–mRNA ceRNA networks, wide host responses to viral infection were enriched. Viral replication regulation is one of the most important events in EHI and may impact the long term clinical outcome of patients; therefore, we further focused on circRNAs associated with viral replication regulation through circRNA–miRNA–mRNA ceRNA network analysis. Firstly, 3 mRNAs, CCNK, CDKN1A and IL-15, reported to be involved in HIV-1 replication that were differentially expressed in our RNA-Seq data were selected, among which 5 DE miRNAs were predicted to have MRE binding sites, including miR-27b-3p, miR-542-3p, miR-101-3p, let-7c-5p and miR-548ah-3p. A total of 67 DE circRNAs that were expressed above the median level were predicted to have shared MREs with CCNK, CDKN1A and IL-15 and were determined to be potentially related to HIV-1 replication. Among them, 18 circRNAs (2 with the ‘8mer’ sequence match and 16 with the ‘7mer’ sequence match between circRNAs and miRNAs) were predicted to share MREs of miR-27b-3p with CCNK (Table 1 and Additional file 4: Table S4) and might act as ceRNAs of miR-27b-3p targeting CCNK. Another 40 circRNAs (12 with the ‘8mer’ sequence match and 28 with the ‘7mer’ sequence match between circRNAs and miRNAs) were predicted to share MREs of miR-542-3p, miR-101-3p and let-7c-5p with CDKN1A, suggesting the ceRNAs of the above miRNAs might be targeting CDKN1A (Table 1 and Additional file 4: Table S4). In addition, 9 circRNAs (4 with the ‘8mer’ sequence match and 5 with the ‘7mer’ sequence match between circRNAs and miRNAs) were predicted to be ceRNAs of miR-548ah-3p targeting IL-15 (Table 1 and Additional file 4: Table S4).

**Table 1:** circRNA-associated-ceRNA networks predicted to be involved in HIV-1 replication

mRNAs	miRNAs	Seed match	circRNAs
CCNK	miR-27b-3p	8mer	chr7:26236021−26237352−
		8mer	chr6:31236599−31321721−
CDKN1A	miR-542-3p	8mer	chr13:45112671−45114072−
		8mer	chr13:42385361−42393522−
		8mer	chr14:68151732−68157138−
		8mer	chr15:43440953−43452992+
		8mer	chr5:82832826−82838087+
		8mer	chr6:31236599−31321721−
CDKN1A	miR-101-3p	8mer	chr6:32487147−32548633−
		8mer	chr1:38052912−38054683−
CDKN1A	let-7c-5p	8mer	chr13:45112671−45114072−
		8mer	chr20:45891032−45912392−
		8mer	chr12:69210592−69218431+
		8mer	chr16:354304−364683−
IL15	miR-548ah-3p	8mer	chr16:19656208−19663412+
		8mer	chr8:48866180−48869991−
		8mer	chr15:94910834−94928754+
		8mer	chr15:55516087−55527154−

## Discussion

In this study, we systematically analyzed circRNA, miRNA, and mRNA profiles and constructed a circRNA-associated-ceRNA network among PBMCs from HARRT-naive EHI patients. We found EHI significantly altered the circRNA expression profile, with 1365 DE circRNAs identified to be differentially expressed during EHI. The basic characters of the DE circRNAs were largely in accordance with other diseases and cell types in chromosomal distribution, categories and length [48, 49]. In our study, we found the number of up-regulated circRNAs (912/1365) was higher than that of down-regulated circRNAs (453/1365), and this result is in accordance with the circRNA profiles in some other diseases and cell types, such as in radioresistant esophageal cancer cells [50], but is different than the circRNA profiles in 7-month-old senescence accelerated mouse prone 8 (SAMP8) model brains [51] and other diseases [52].

In this study, the potential functions of candidate circRNAs and the circRNAs associated biological processes in EHI were predicted through circRNA-associated-ceRNA network analyses. According to our results, multiple DE circRNAs were found to be involved in immune response, inflammatory response, and defense responses to viral infection, which have been reported to play important roles in the pathogenesis and disease progression of HIV-1 infection [53, 54]. Since HIV-1 infection cannot be cured with current antiretroviral therapy (ART), additional efforts are needed to elucidate how to use host defense responses against HIV-1 infection; moreover, inflammatory responses cannot be completely restored even on ART [55], suggesting the mechanisms of HIV pathogenesis are still not fully understood.

This study provided more clues for understanding the mechanisms of HIV pathogenesis in EHI through circRNA-associated-ceRNA networks. To our knowledge, no prior research has reported on the roles of circRNAs in EHI; however, some studies have revealed a role of circRNAs involved in tumor immunity regulation and immunotherapy. For example, circFoxo3 was reported to regulate immune responses during tumor development by modulating some proteins like p53 [56, 57]. Furthermore, exogenous circRNA was reported to co-aggregate with RIG-I, which could sense exogenous circRNAs and stimulate the expression of innate immune genes [34]. Innate immunity is very important in HIV replication control in EHI [3], and in this study, we predicted circRNAs may act as ceRNAs to regulate the expression of innate immune genes such as OAS1, OAS2, and OAS3 (Fig. 3), which have previously been reported to act as antiviral factors through catalyzing the synthesis of 2′-5′-linked oligoadenylates, leading to the activation of RNAse L and degradation of viral and cellular RNAs [58, 59]. In summary, this study showed that circRNAs might play an important role in HIV pathogenesis, targeting circRNAs that might regulate the host response against HIV-1.

As we all known, the balance between virus turnover and host immune responses maintains the viral set point in the absence of ART [2]. The viral set point is a crucial determinant of HIV-1 disease progression. Some genes have been reported to regulate HIV-1 replication and impact the viral set point level. In this study, we found 3 series of circRNAs potentially involved in HIV replication regulation through a ceRNA network. The first was a 40 circRNA/miR-542-3p/miR-101-3p/let-7c-5p/CDKN1A regulatory network. CDKN1A, also named P21, is a documented p53 downstream gene restricting HIV-1 replication both dependently and independently of the HIV-1 restriction factor SAMHD1 [60–62]. In this study, we found that miR-542-3p, miR-101-3p and let-7c-5p might target both CDKN1A and 40 circRNAs. Moreover, the expression level of the above miRNAs and circRNAs were inversely regulated in EHI, suggesting that these circRNAs might regulate the expression of CDKN1A via acting as miRNA sponges and contributing to HIV-1 replication regulation. The second was the 18 circRNA/miR-27b-3p/CCKN regulatory network. miR-27b has been reported to regulate HIV-1 replication and the expression of cyclin T1 in resting CD4 T cells, cyclin T1 is a regulatory subunit of a positive RNA polymerase II transcription elongation factor also known as P-TEFb and is also needed for Tat transactivation of HIV-1 gene expression [63]. CCNK, also named Cyclin K, was found to displace cyclin T1 from the P-TEFb complex by utilizing HIV-1 Nef protein, resulting in the inhibition of HIV-1 gene expression and replication [64]. This study revealed 18 circRNAs may function as miRNA sponges of miR-27b to regulate HIV-1 replication via regulating the expression of CCNK. The third was the circRNA/miR-548ah-3p/IL-15 regulatory network. Mueller et al. [65] had previously reported that IL-15 treatment during EHI increased the viral set point and accelerated disease progression in an animal model, suggesting an effect of IL-15 on viral replication. In our ceRNA network, chr16:19656208−19663412+ and another 8 circRNAs shared MERs with IL-15 and might act as ceRNA of miR-548ah-3p and, therefore, potentially impact the viral set point by regulating IL-15. We speculated that deregulation of these circRNAs might be related to HIV-1 replication of EHI, and this provides a novel direction for study of the mechanisms of HIV-1 replication.

There are also some limitations in this research. First, total PBMCs were used for RNA-Seq and miRNA-Seq in this study, and thus, the results only represent the most prominent or common biological processes in EHI. Although CD4+ T cells, major targeting cells of HIV infection, are one of the major cells included in PBMCs, only a small fraction of CD4+ cells are infected with HIV, and further studies are needed to uncover the biological processes in specific cell types and in infected cells. Second, the standard library protocol that we used in RNA-Seq would lead to strand-agnostic RNA-Seq data, which is difficult to discern antisense RNA biology compared to a strand specific library protocol [66, 67]. Third, the sample size of this study is relatively small because of limitation of RNA sample amount, and further studies are necessary to provide a larger sample size to verify our findings.

## Conclusions

This study, for the first time, identified a circRNA profile and predicted potential circRNAs involved in mechanisms of pathogenesis in EHI, especially in the viral replication regulation, which provides novel targets for further research into both the molecular mechanisms of EHI and the potential targets of HIV infection treatment.

## Additional files

**Additional file 1: Table S1.** Clinical characteristics of participants.

**Additional file 2: Table S2.** Gene ontology analyses of the DE mRNAs.

**Additional file 3: Table S3.** Differentially Expressed miRNAs.

**Additional file 4: Table S4.** circRNA-associated-ceRNA networks that are most likely involved in HIV-1 replication.

## Abbreviations

HIV-1: human immunodeficiency virus 1
AIDS: acquired immunodeficiency syndrome
EHI: early HIV infection
circRNA: circular RNA
ceRNA: competing endogenous RNA
miRNA: microRNA
lncRNA: long non-coding RNA
PBMC: peripheral blood mononuclear cell
HC: healthy control
RNA-Seq: RNA sequencing
miRNA-Seq: miRNA sequencing
GO: gene ontology
MRE: miR response element
ART: antiretroviral therapy
CDKN1A: cyclin-dependent kinase inhibitor 1A
RT-qPCR: real-time quantitative PCR
